# Comparable level of aggression between patients with behavioural addiction and healthy subjects

**DOI:** 10.1038/s41398-021-01502-8

**Published:** 2021-07-05

**Authors:** Yui Asaoka, Moojun Won, Tomonari Morita, Emi Ishikawa, Yukiori Goto

**Affiliations:** 1 Primate Research Institute, Kyoto University, Inuyama, Aichi 484-8506 Japan; 2 Kyowa Hospital, Obu, Aichi 474-0071 Japan; 3 Present Address: MRC Lab Clinic, Mitaka, Tokyo 181-0012 Japan

**Keywords:** Addiction, Human behaviour

## Abstract

Heightened aggression is identified in several psychiatric disorders, including addiction. In this preliminary study with a relatively small number of samples, aggression in subjects diagnosed with behavioural addiction (BA) was implicitly assessed using the point subtraction aggression paradigm (PSAP) test along with measurements of oxy- and deoxyhaemoglobin dynamics in the prefrontal cortex (PFC) during the test using functional near-infrared spectroscopy. Aggression in BA patients was no higher than that of healthy control (CT) subjects in the PSAP test. Although no apparent increase or decrease in haemoglobin concentrations was observed in the PFC of either BA patients or CT subjects, abnormal correlations within the PFC network were present in BA patients. Consistent with comparable aggression between the groups, blood concentrations of the sex hormone testosterone, which has been shown to be associated with aggressiveness, was even lower in BA patients than in CT subjects. In contrast, when a set of questionnaire surveys for the assessment of aggression were administered, BA patients rated themselves as more aggressive than non-BA subjects. Collectively, these results suggest that aggression may not be heightened in BA, but BA patients may overestimate their aggressiveness, raising concerns about the use of questionnaire surveys for assessments of affective traits such as aggression in behavioural addiction.

## Introduction

Kleptomania (KM) and paraphilia (PP), which are formally diagnosed as impulse control disorders in the Diagnostic and Statistical Manual of Mental Disorders 5th Edition (DSM-5) [1], have been suggested to meet the criteria of behavioural addiction (BA) and therefore are considered interchangeable in this category of disorders [2–5]. These psychiatric conditions involve symptoms of addiction, such as repetitive and persistent engagement and loss of control over specific behaviours despite negative consequences [2–5]. Currently, a major challenge of BA is that, with the exception of pathological gambling, there is a relatively limited number of studies and insufficient evidence to include some impulse control disorders, such as KM and PP, in addition to the disorder in the diagnostic manuals [5].

Heightened aggression has been reported in psychiatric disorders, including addiction [6–9]. One BA is intermittent explosive disorder, which is characterized by impulsive and angry outbursts resulting in violence and aggression towards others [10]. Some BAs, such as Internet addiction and gaming disorder, have also been associated with increased engagement in aggressive behaviour [6–8], although aggression in other BA symptoms has been less explored [9]. Studies have demonstrated that aggression can be divided into subcategories, such as proactive vs. reactive aggression, which are cold (absence of provocation or anger and goal-directed) and hot (anger and rage, autonomic arousal, and loss of impulse control) forms of aggression, respectively [11]. The way that each of these subcategories of aggression is associated with BA has remained elusive.

Several tools have been developed to assess aggression in human subjects. A commonly utilized approach is questionnaire surveys. There are various types of questionnaires to assess aggression, such as the Buss–Perry aggression questionnaire (BP-AQ) [12], which examines four different traits of aggression, i.e., physical aggression, verbal aggression, anger, and hostility, and the reactive-proactive aggression questionnaire (RPQ) [13], which examines proactive and reactive aggression. These questionnaire surveys address self-referential aggression. In addition, aggression can be implicitly assessed using a psychological test, such as the point subtraction aggression paradigm (PSAP) test [14,15,], in which subjects engage in the test without knowing that the test aims to examine aggression. Aggression can also be evaluated indirectly by measuring blood concentrations of the sex hormone testosterone in both male [16] and female [17] subjects, although meta-analysis studies find that a correlation between testosterone and aggression is generally weak at best [18–20] and that many confounding factors, such as the stress hormone cortisol, are also involved [21,22,].

Collectively, previous studies suggest that aggression is heightened in BA [6–8]. However, whether such heightened aggression is mutually observed among different dimensions of BA symptoms remains unclear given that studies investigating aggression in BA have been limited to specific aspects of BA symptoms, such as Internet and gaming disorder [6–8], and no studies to date have examined patients with other BA symptoms, such as KM and PP. If heightened aggression is found to be a common feature of BA, behavioural therapy to manage aggression and violent behaviours, such as aggression control therapy [23], could be an important clinical approach for the treatment of BA.

In this preliminary study, we investigated whether aggression might be heightened in a relatively small number of patients diagnosed primarily with KM and PP compared to healthy subjects. We particularly evaluated aggression with a method distinct from previous studies [6–9]. Heightened aggression was reported in BA using questionnaire surveys by administering the PSAP test for implicit assessment along with an investigation of neural activity in the prefrontal cortex (PFC) using functional near-infrared spectroscopy (fNIRS) during the test to further evaluate the neural correlates of aggression since previous studies have also reported that PFC activity is associated with the expression of aggression [24–26]. Aggression was further examined with two additional approaches, one with appraisals of blood testosterone concentrations and the other with a set of questionnaire surveys for aggression. We hypothesized that similar to patients with other BA symptoms, KM and PP patients might exhibit signs of heightened aggression in these study measures. Thus, BA patients might self-report being more aggressive in the questionnaire surveys and exhibit higher aggression in the PSAP test along with altered PFC activity and higher blood testosterone concentrations than healthy subjects.

## Subjects and methods

### Participants

This study was conducted in accordance with the Declaration of Helsinki and the Ethical Guidelines for Medical and Health Research Involving Human Subjects by the Japanese Ministry of Health, Labour and Welfare. All experimental procedures were approved by the Human Research Ethics Committee of Kyoto University Primate Research Institute and the Ethics Committee of Kyowa Hospital. Experiments were conducted only once, and no replication was attempted, in each participant in this study.

As a control (CT) group, a total of 31 healthy subjects (36.2 ± 2.00 years old; 13 males, 18 females) who were working staff of Kyowa Hospital (*n* = 25) and students at Kyoto University (*n* = 6) were recruited. The inclusion criteria were subjects older than 18 years and without smoking and psychiatric histories. These CT subjects were recruited by advertisements. The BA group consisted of hospitalized patients who were diagnosed with BA (*n* = 16; 39.6 ± 3.68 years old; 9 males, 7 females) with different symptoms, i.e., gambling (GA; *n* = 1), KM (*n* = 10) and PP (*n* = 5). Upon enrolment in the study, written informed consent was obtained from all participants in advance of the experiments. Patients were diagnosed by a psychiatrist (Moojun Won, M.D.) who has specialized in addiction treatment for more than 22 years. Patients were diagnosed based on the criteria of the DSM-5 [1] and ICD-10 [27]. The severity levels of patients were judged using the Japanese version of the addiction severity index [28]. The study was conducted among these patients at the time of hospitalization and before clinical treatments were started during the period between May 2019 and February 2020. BA patients were recruited by the attending physician at the time of hospitalization. The inclusion criteria were subjected older than 18 years who were diagnosed with BA but no other psychiatric conditions. In the study period, BA patients with KM, PP, and GA but no other BA symptoms were hospitalized, so the BA group in this study consisted only of KM, PP, and GA. The exclusion criteria for both CT and BA subjects were the inability to understand the instructions on the administered tests or an estimated full-scale intelligence quotient (IQ) below 60 with the short form of the Japanese version of the Wechsler Adult Intelligence Scale (WAIS)-III [29,30,]. No difference in the sex ratio (Fisher’s exact test, *p* = 0.376) or age (unpaired *t*-test, *p* = 0.375) was observed between the BA and CT groups. The BA group consisted of heterogeneous symptoms (KM, PP, and GA) that were analysed together in this study since (i) no difference was observed between KM and PP patients in measurements of cognitive function [31], negative affect such as stress and anxiety [32], and blood monoamine concentrations [33] in our past studies, and (ii) no difference was observed between KM and PP patients in any measurements of aggression in the current study.

### PSAP test

Aggression was examined by computer-based administration of the PSAP test. The PSAP test was employed in this study as this test has been demonstrated to reliably assess aggression in various subjects with and without psychiatric conditions [14,15, 34–36]. Moreover, the test is designed to implicitly assess aggression and therefore assesses the level of aggression independent of intentional control of the expression of aggression by subjects. The test was programmed and delivered using Inquisit Lab software (Millisecond Software, Seattle, WA, USA). At the beginning of the test, participants were instructed to earn as many points as possible, which were later exchanged for monetary rewards, by competing against an opponent. Participants were told that the opponent was a real human counterpart who was playing the game over the Internet, although the opponent was, in fact, imaginary and computer-programmed. In the test, three choices, “A”, “B”, and “C”, were presented on the screen, and participants chose one of them by pressing the keyboard button corresponding to each of the choices on the screen. By pressing the “A” button 100 times, participants could earn 1 point, which they were told equalled 50 Japanese yen at the end of the experiments. Participants were told that the imaginary opponent could steal their earned points at any point in the game, which was set by computer programming at random intervals varying from 3 to 60 s. Participants were also given two other options: to penalize the imaginary counterpart by subtracting points by pressing the “B” button, with 10 presses of the button resulting in 1 point subtracted from the opponent, or to protect their earned points from the opponent during an uncertain interval by pressing the “C” button 10 times. Participants were explicitly informed that a penalty to the opponent by selecting the “B” button was not credited to the participants themselves as earned points. The participants repeated choosing the A, B, or C button for 10 min. The number of times the B button was chosen throughout the test was considered the level of aggression.

### fNIRS

PFC activity associated with the PSAP test was examined with fNIRS. NIRO-200 NIRS Image Processing and Measuring System (Hamamatsu Photonics K.K.) was used in this study. Two emitters (delivering laser pulses at wavelengths of 775, 810, and 850 nm) and 8 detectors with the distance between an emitter and a detector at 3.0 cm enabled 10 points of measurements over the PFC, spanning the left and right Brodmann areas 6, 8, 9, 45, and 46. Oxygenated (oxy-Hb) and deoxygenated (deoxy-Hb) haemoglobin concentrations at a sampling rate of 0.5 Hz were recorded. Since prolonged oxy-Hb and deoxy-Hb concentration changes were observed over the test, the area under the curve (AUC), i.e., summations of oxy-Hb and deoxy-Hb changes over time, was calculated at each recording site, and subsequent data analysis was conducted with the AUC.

### Testosterone assay

Blood testosterone concentrations were measured using enzyme-linked immunosorbent assay (ELISA) to indirectly assess aggression. Whole blood samples were collected from BA patients around noon (11:00–11:30 a.m.) and from CT subjects in the afternoon (15:00–17:00) one day prior to or on the day of the psychological tests, depending on the schedules of supporting staff in the hospital. Samples were stored in the freezer at −30 °C until the days of processing for ELISA. Sample processing was conducted using the commercially available human ELISA testosterone assay kit from Bioassay Technology Laboratory (Cat. No. E1036Hu) according to the user manual. After processing, ELISA plates were read using an iMark microplate reader (Bio-Rad, Hercules, CA).

### Questionnaires

Two paper-based questionnaires, BP-AQ [12] and RPQ [13], were administered to evaluate self-referential reports of aggression. These two questionnaires were selected and administered since the BP-AQ, along with its translation into non-English languages, has been used extensively in various studies, demonstrating that it can reliably assess self-awareness of aggression independent of the cultural backgrounds of subjects [37,38,]. In addition, given that aggression can be divided into different dimensions (proactive vs. reactive [13]) that cannot be assessed with the BP-AQ, the RPQ was also administered to further examine the associations between aggression subcategories and BA. The BP-AQ comprises 29 items. A total score indicates trait aggression, and the score is divided into four subscales: physical aggression, verbal aggression, anger, and hostility. The RPQ assesses two categories of aggression: proactive aggression, which is aggression without emotion that is cold-blooded, thought out, instrumental, and motivated by external rewards, and reactive aggression, which is aggression motivated by perceived or actual provocation due to hostile attribution bias. The RPQ consists of 23 items, with 11 and 12 items assessing reactive and proactive aggression, respectively. A summed score provides a measure of general aggression.

### Data analysis

Investigators who were not blinded to the experimental conditions collected the data and performed statistical analyses. All statistical analyses were conducted using Statistica software (StatSoft, Tulsa, OK, USA). A probability value of *p* < 0.05 was considered statistically significant. Normality in sample distributions was evaluated with Shapiro–Wilk’s *W* test. In addition, Levene’s test was conducted to evaluate the homogeneity of variances between groups. When parameters were found not normally distributed or variances were significantly different between the groups with these tests, a nonparametric statistical method was used for comparisons between groups. Accordingly, an unpaired *t*-test or Mann–Whitney *U* test was used for comparisons between BA and CT or between KM and PP. Linear correlation analysis was employed to examine the correlations between implicit and explicit measurements. Since the sample size was relatively small in this study (*n* = 16 for BA patients) and some analyses were highly sensitive to individual data points, outliers were carefully assessed using the median absolute deviation (MAD) method, which is suggested to be more appropriate for detecting outliers than the ordinal, mean plus/minus three standard deviations method [39]. This analysis found that none of the data points reached the criterion to exclude outliers. The datasets generated and analysed during the current study are available from the corresponding author upon reasonable request.

## Results

### Assessments of aggression with the PSAP test

The PSAP test was conducted, along with fNIRS recordings of PFC activity, among the BA patients and CT subjects. The number of choices to earn points (A button), to attack the imaginary opponent (B button; aggression), and to protect from attacks by the opponent (C button) at the expense of opportunities to earn points in the PSAP test were recorded.

**Fig. 1:**
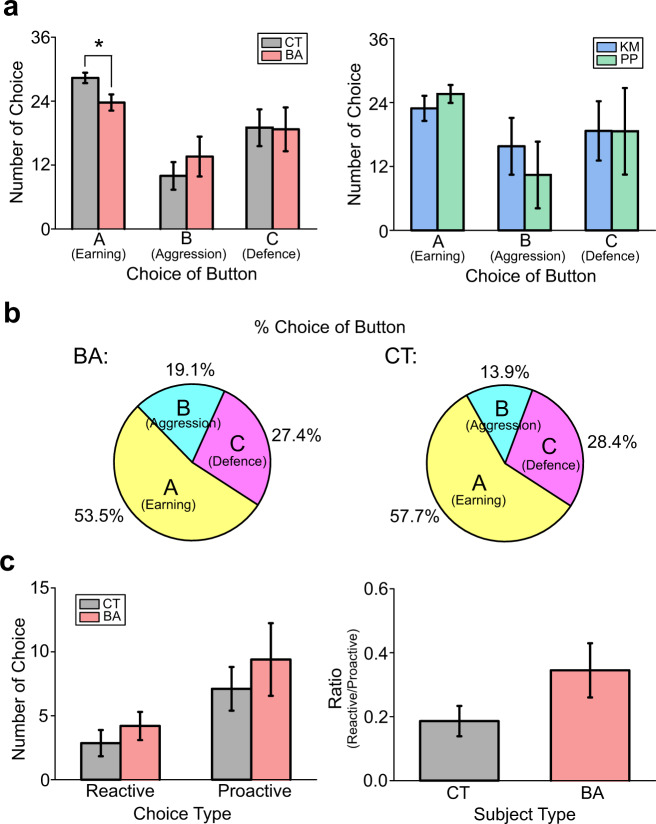
Assessments of aggression with the PSAP test. **a** Bar graphs illustrating the number of choices for earning points (A button), attacking the opponent (B button), and protecting point subtractions (C button) in behavioural addiction (BA) patients and control (CT) subjects (left) as well as in kleptomania (KM) and paraphilia (PP) patients (right). Graphs show the means with error bars indicating s.e.m. *p < 0.05. **b** Pie charts illustrating percentages of choices for the A, B, and C buttons relative to the total choices in BA patients (left) and CT subjects (right). (**c**) Graphs showing the number of reactive and proactive aggression choices (left) and the ratio of reactive to proactive aggression choices (right) in BA patients and CT subjects.

The number of choices to earn points was significantly lower in BA patients than in CT subjects (unpaired *t*-test, *t*_41_ = −2.68, *p* = 0.011; Fig. 1a), whereas the number of choices to attack the imaginary opponent and the number of choices to protect point subtractions did not differ between BA patients and CT subjects (Mann–Whitney *U* test *Z* = 1.01, *p* = 0.314; Fig. 1a). There was no difference in any of the choices between KM and PP patients (Fig. 1a). No differences were found between BA patients and CT subjects even when the number of choices for each category was converted into percentages relative to all choices (Fig. 1b).

The number of choices to attack was further evaluated by dividing the choices into reactive and proactive aggression in response to point subtractions by the imaginary opponent (choosing to attack immediately after point subtractions) and those spontaneously made without point subtractions. No statistically significant difference was observed in the number of reactive or proactive aggression choices between BA patients and CT subjects (Fig. 1c), although the ratio of reactive to proactive aggression choices tended to be higher in BA patients than in CT subjects (*t*_41_ = 1.78, *p* = 0.083; Fig. 1c).

These results suggest that, in contrast to previous studies reporting heightened aggression in addiction patients, aggression may be comparable between BA patients and CT subjects when aggression is implicitly assessed with the PSAP test.

### PFC activity associated with the PSAP test

**Fig. 2:**
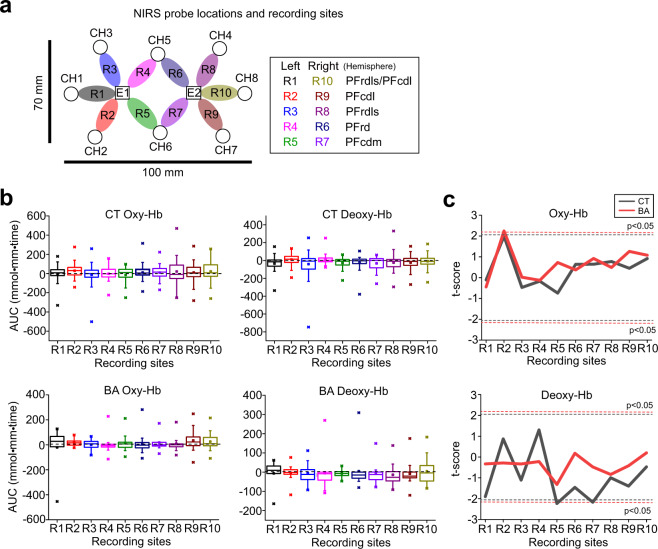
Oxy-Hb and deoxy-Hb changes in the PFC associated with the PASP test. **a** A schematic diagram illustrating the probe (emitters = E1–2, detectors = CH1–CH8) locations and the recording sites (R1–R10) on the skull. **b** Graphs showing oxy-Hb and deoxy-Hb changes during the PSAP test at each recording site in behavioural addiction (BA) patients and control (CT) subjects. **c** Graphs showing *t*-scores of oxy-Hb (top) and deoxy-Hb (bottom) changes at each recording site. Dashed lines indicate the thresholds above or below which *t*-scores are statistically significant.

Given that PFC cognitive control plays an important role in the regulation of aggression with lower PFC activity associated with heightened aggression [24–26], we investigated oxy-Hb and deoxy-Hb changes in the PFC with fNIRS during the PSAP test (Fig. 2a).

**Fig. 3:**
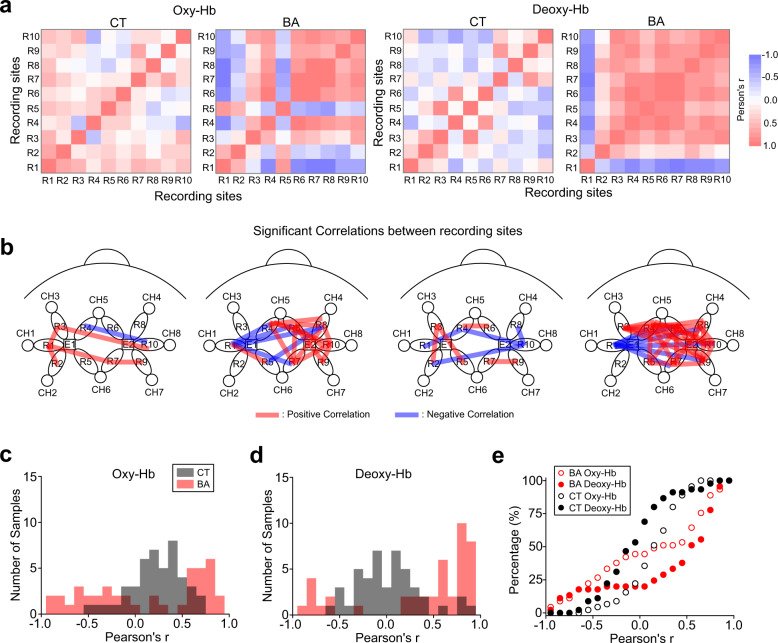
Correlations of Oxy-Hb and deoxy-Hb changes within the PFC network associated with the PASP test. **a** Heat maps illustrating correlations (Pearson’s *r*) of oxy-Hb and deoxy-Hb changes in every possible pair of recording sites. BA behavioural addiction, CT control. **b** Schematic diagrams illustrating correlations in pairs of recording sites that were statistically significant (*p* < 0.05). Positive and negative correlations are illustrated with red and blue lines, respectively. **c**–**e** Histograms illustrating distributions of Pearson’s *r* for oxy-Hb (**c**) and deoxy-Hb (**d**) and a cumulative histogram (**e**) in BA patients and CT subjects.

Consistent with the lack of differences in performance related to aggression in the PSAP test, oxy-Hb and deoxy-Hb changes were neither higher nor lower in BA patients than in CT subjects (Fig. 2b, c). However, the correlations (Pearson’s *r*) of oxy-Hb and deoxy-Hb changes in pairs of recording sites were substantially different between BA patients and CT subjects (Fig. 3a–e). The correlations of oxy-Hb and deoxy-Hb changes in the pairs of CT subjects attained normal distributions around *r* = 0.3 and *r* = 0, respectively, in the histograms (Fig. 3c–e), suggesting that there were weak correlations of oxy-Hb changes, but not deoxy-Hb changes, in several pairs of the recording sites in CT subjects. In contrast, bimodal distributions with peaks higher than *r* > 0.5 and lower than *r* < −0.5 in the histograms were observed in BA patients, suggesting that oxy-Hb and deoxy-Hb changes in a substantial number of pairs of recording sites were abnormally correlated either positively or negatively in BA patients (Fig. 3c–e)

These results suggest that PFC connectivity may be compromised in BA patients in the PSAP test, although such neuronal alterations may not be associated with aggression but with other aspects of task performance.

### Testosterone concentrations in blood samples

To further investigate aggression in BA patients and CT subjects, we examined basal blood testosterone concentrations in samples obtained from the participants.

**Fig. 4:**
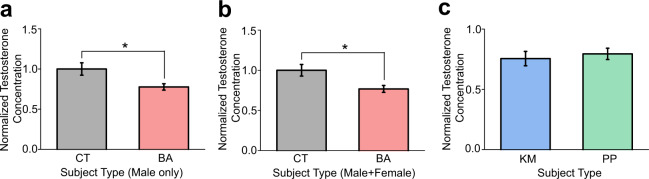
Blood testosterone concentration assays with ELISA. **a** Bar graph showing testosterone concentrations in blood samples from male behavioural addiction (BA) patients and control (CT) subjects (top). **b** A graph similar to (a) but showing BA patients and CT subjects with both male and female subjects combined. **c** **A** graph similar to (**a**) but showing KM and PP patients.

Basal testosterone concentrations in blood samples obtained from male BA patients (*n* = 9) were significantly lower than those of male CT subjects (*n* = 9; *t*_16_ = −2.58, *p* = 0.020; Fig. 4a). Lower testosterone concentrations were retained even when both male and female samples were combined (BA patients, *n* = 16; CT subjects, *n* = 24; *t*_38_ = −2.43, *p* = 0.020; Fig. 4b). Testosterone concentrations did not differ between KM and PP patients (Fig. 4c).

These results suggest that, consistent with the lack of heightened aggression in BA patients in the PSAP test, blood testosterone concentrations are comparable or even lower in BA patients than in CT subjects.

### Questionnaire surveys for aggression

To investigate the reason for the discrepancy between heightened aggression reported in previous studies and the failure to find such heightened aggression in BA patients in the current study, we administered two questionnaire surveys, the RPQ and BP-AQ, for the self-rating of aggression.

**Fig. 5:**
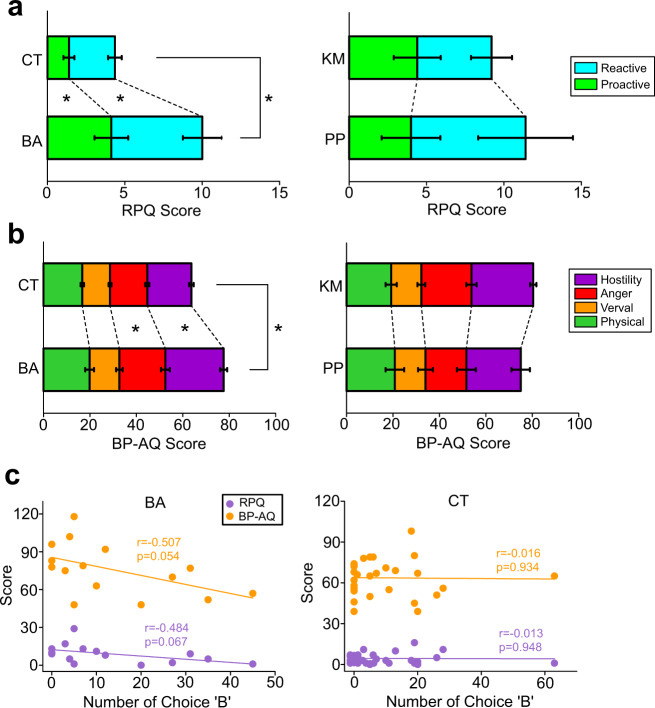
Questionnaire surveys for assessments of aggression. **a** Bar graphs showing comparisons of proactive vs. reactive aggression with the Reactive-Proactive Aggression Questionnaire (RPQ) between behavioural addiction (BA) patients and control (CT) subjects (left) and between kleptomania (KM) and paraphilia (PP) patients (right). **b** Graphs similar to (a) but showing assessments of aggression with the Buss–Perry Aggression Questionnaire (BP-AQ), in which scores for physical aggression, verbal aggression, anger, and hostility dimensions of aggression were stacked. (**c**) Graphs showing correlations between the number of choices reflecting aggression (B button) and the RPQ and BP-AQ scores in BA patients (left) and CT subjects (right).

In the RPQ, BA patients rated higher scores than CT subjects overall (*Z* = 2.92, *p* = 0.003; Fig. 5a). Further analysis revealed that scores for both proactive (*Z* = 2.69, *p* = 0.007) and reactive (*Z* = 1.96, *p* = 0.049) aggression were significantly higher in BA patients than in CT subjects. There was no difference in the scores of this questionnaire between KM and PP patients (Fig. 5a).

In the BP-AQ, BA patients also rated higher scores than CT subjects overall (*Z* = 2.83, *p* = 0.005; Fig. 5b). Further analysis revealed that the anger (*Z* = 3.64, *p* < 0.001) and hostility (Z = 2.97, *p* = 0.002) scores were significantly higher in BA patients than in CT subjects, but this was not the case for other traits (physical aggression and verbal aggression; Fig. 5b). There was no difference in the scores of this questionnaire between KM and PP patients (Fig. 5b).

In contrast to the lack of indication of higher aggression in BA patients in the PSAP test, aggression scores were higher in BA patients than in CT subjects in these questionnaires. Thus, correlations between the scores of the questionnaires and the number of attacks in the PSAP test were analysed. The number of attacks in the PSAP test that BA patients exhibited, although statistically not significant, showed trends of negative correlations with the scores of both the RPQ (*r* = −0.484, *p* = 0.067; Fig. 5c) and the BP-AQ (*r* = −0.507, *p* = 0.054; Fig. 5c). In contrast, no correlation was observed in CT subjects (Fig. 5c).

These results suggest that although aggression is comparable between BA patients and CT subjects when examined implicitly, BA patients rate themselves as more aggressive than CT subjects in self-referential assessments, which may be due to aberrant self-evaluation.

## Discussion

In this preliminary study with a relatively small sample size, contradictory to our hypothesis, we found that aggression is comparable between BA patients and CT subjects. However, in regard to the questionnaire surveys, BA patients rated themselves as more aggressive than CT subjects. At the neuronal level, PFC activity appears to be compromised during the PSAP test in BA patients, although these altered PFC activity patterns may not be associated with aggression.

There are several major limitations of this study. The most important limitation is the sample size; therefore, this study should be considered preliminary. Sixteen BA patients with heterogeneous symptom categories were recruited in this study. Confirmation and replication of the results in the current study will be required with a larger sample size with homogeneous symptoms. In particular, the smaller sample size could be the reason for failure to find a difference in aggression between the BA and CT groups in the PSAP test. However, the effect size [40,41,] was 0.856 for earning points, which is a sufficiently large effect size according to Cohen [42], whereas the effect size for aggression was only 0.260. With this small effect size, which is near the cut-off range for a trivial difference of the means for aggression, it is less likely to find a significant difference even if the sample size is increased. Power analysis also found that the estimated number of samples required to find a difference at 95% confidence (*p* = 0.05) was *n* = 309 for each group. This sample size of more than 300 is far larger than the sample sizes of any other PSAP studies that found detectable differences in aggression between groups (the study with the largest sample number to date was approximately *n* = 100 in each group, and most studies were conducted with fewer than *n* = 50 [43]). Therefore, even if the sample size is increased and a larger sample size reveals a difference, it is likely to be extremely small and negligible compared to other studies. In this study, we also found no difference between KM and PP patients in any measurements of aggression, which is consistent with our past studies demonstrating no difference between KM and PP patients in cognitive function [31], negative affect [32], and blood monoamine concentrations [33], although the whole-genome methylation analysis has revealed that DNA methylation status on 187 CpG sites differed between KM and PP patients [33]. Thus, further study is required to compare KM and PP patients in more detail with a larger sample size.

Study measures were administered to BA patients at the time of hospitalization. Hospitalized BA patients were admitted for clinical treatment of BA as a part of criminal sentencing. In addition, being detained in a hospital may be distressing for some patients. These situations could cause stress in BA patients, which in turn affects aggression. We previously found that these BA patients were more stressed than CT subjects [32]. This heightened negative affect could be a confounding factor as stress has been shown to reinforce aggression and vice versa to create a vicious cycle, at least in rodents [44]. However, this is unlikely since our finding contradicts the prediction. Thus, even though BA patients were more stressed, they exhibited comparable, but not heightened, aggression with that of CT subjects. In addition, most CT subjects were working staff of Kyowa Hospital (*n* = 25 out of 31) where the BA patients were hospitalized; therefore, the study measures were conducted in an identical room for both BA patients and CT subjects, further excluding the possibility of environmental influence on the study measures.

Aggression was assessed implicitly with the PSAP test. Given that aggression can be divided into proactive and reactive aggression [11], we further evaluated the number of aggressive choices separately for choices made in response to the point subtraction by the imaginary opponent and those without it, which are thought to correspond to proactive and reactive aggression, respectively. However, there was no difference in any of these measurements between BA patients and CT subjects. Higher aggression in the PSAP test has been reported in abusers of addictive substances, such as heroin [45], methamphetamine [46], alcohol [47], and cannabis [47]. However, another study has shown that addictive substance abusers with comorbid antisocial personality disorder exhibit aggression comparable to that of healthy subjects in the PSAP test, yet they still report themselves to be more aggressive in a questionnaire such as the BP-AQ [48]. Antisocial personality disorder patients without substance abuse also exhibit no heightened aggression in the PSAP test [49] but report themselves to be more aggressive than healthy subjects in the questionnaire [50]. Thus, the findings in this study may be more consistent with the characteristics of antisocial personality disorder than drug addiction. Although no BA patients in our study were diagnosed with antisocial personality disorder, their primary symptoms were KM and PP, which are considered antisocial behaviours. Further investigation with the PSAP test in patients with other BA symptoms, such as gambling disorder, Internet addiction, and gaming disorder, which are less relevant to antisocial behaviours, would yield insight on this issue.

We conducted blood testosterone assays using ELISA as an indirect assessment of aggression and found that testosterone concentrations were even lower in BA patients than in CT subjects. Although testosterone has been associated with aggression in both male [16] and female [17] subjects, several confounding factors influence this association; therefore, high or low testosterone does not directly result in high or low aggression. In particular, the stress hormone cortisol has been demonstrated to be one of the major confounding factors [22]. Human studies have shown that low basal cortisol is correlated with heightened trait aggression [51]. Aggression is also correlated with serum testosterone, but this is viable under low serum cortisol conditions [52], suggesting the “high testosterone/low cortisol” model for heightened aggression [22]. In this study, we found that plasma testosterone was lower in BA patients than in CT subjects. Moreover, in our previous study, we found that plasma cortisol was higher in BA patients than in CT subjects [32]. Thus, the lower than normal testosterone in BA patients observed in the current study is consistent with previous literature demonstrating that cortisol administration decreases testosterone in both animals [53] and humans [54]. Low testosterone and high cortisol are the hormonal features of BA patients. There are now at least a handful of studies that have investigated aggression in subjects with low testosterone and high cortisol conditions [21,52, 55–63]. None of the studies, regardless of different backgrounds of the subjects, found higher aggression in these subjects (although lower aggression has been reported). Therefore, based on these hormonal profiles, it is conceivable that aggression in the observed BA patients in the current study may not be heightened even with other confounding factors, such as sex, personality, and clinical diagnosis [22, 64–66]. The lack of heightened aggression in BA is further supported by our previous study showing that the plasma concentration of the serotonin metabolite 5-hydroxyindole acetic acid (5-HIAA) and the turnover of serotonin (5-HT), i.e., the ratio of 5-HIAA to 5-HT, were comparable between BA patients and CT subjects [33]. 5-HT is another molecule that is suggested to play a substantial role in aggression, evidenced by studies showing that aggression is negatively correlated with 5-HIAA [67], and selective serotonin reuptake inhibitor (SSRI) treatments decrease aggression [68] in patients with personality disorders. Collectively, these hormonal and neurochemical characteristics of BA patients are consistent with the lack of heightened aggression in the PSAP test. There is also a caveat that lower testosterone levels in BA patients than in CT subjects may be partly associated with different timings of blood sampling between the BA patients and CT subjects. Blood sampling was conducted around noon for BA patients, whereas sampling from CT subjects was primarily performed in the afternoon due to technical restrictions (i.e., depending on when staff who could support blood sampling were available). Indeed, daily fluctuations in blood testosterone concentrations have been reported, with high concentrations in the morning and at night but no substantial difference during the daytime [69,70,], so the influence of these daily fluctuations may be limited.

The PFC has been suggested to play an important role in the expression of aggression. For instance, damage to the dorsolateral PFC in humans results in greater expression of aggression [24]. In addition, associations between left dorsolateral PFC activation and experienced anger and aggression have been reported [71]. Similarly, a study has shown that brain structures and functional connectivity in the bilateral PFC are positively associated with proactive aggression [72]. More recently, manipulation of bilateral PFC activity by transcranial stimulation has been demonstrated to reduce intentions to commit aggression in healthy subjects [26], whereas another study with right dorsolateral PFC stimulation revealed no effect on reactive aggression in healthy subjects but an attenuating effect in drug addicts [73]. Based on these studies, lower PFC activity was expected to be observed if BA involved heightened aggression. We investigated this expectation using fNIRS while the subjects engaged in the PSAP test. Consistent with the lack of heightened aggression, no overall oxy-Hb and deoxy-Hb changes were observed throughout the PSAP test in either BA patients or CT subjects. Nevertheless, unusual correlations within the PFC network were noticed in BA patients compared to CT subjects. Such abnormal connections within the PFC network were more prominent in the right PFC than in the left PFC. Since no difference in aggression was observed between the BA and CT groups, whereas the points earned were different between the groups in the PSAP test, if altered intra-PFC connectivity was associated with any behavioural outcomes of task performance, it could be associated with point earnings rather than aggression. Functional neuroimaging studies have shown that the ventromedial PFC (vmPFC), including the orbitofrontal cortex, is also involved in aggression [22,74,]. Accumulating evidence suggests that aggression is associated not with alterations of vmPFC activity but with dysconnectivity between the vmPFC and limbic structures, such as the amygdala [22,74,]. Such vmPFC–amygdala connectivity could not be measured with fNIRS; therefore, whether vmPFC–amygdala connectivity is altered in BA patients remains unknown in this study. Few, if any, studies have directly examined the relationships between intra-PFC connectivity and aggression in healthy subjects and subjects with a clinical diagnosis. However, hypoconnectivity within the PFC network has been reported in subjects with autism spectrum disorder [75,76,], whereas hyperconnectivity within the PFC network is observed in early course and unmediated patients with schizophrenia [77,78,]. Using fNIRS, a study demonstrated that patients with affective disorders also exhibit hypoconnectivity within the PFC network [79]. Heightened aggression is mutually involved in all autism spectrum disorders [80,81,], schizophrenia [82,83,], and affective disorders [84,85,], collectively suggesting that such altered intra-PFC connectivity may be less closely associated with aggression itself since both hyper- and hypoconnectivity within the PFC network are observed in these psychiatric conditions.

Previous studies that investigated aggression with questionnaire surveys have reported heightened aggression in BA [6–8]. Our study is partly consistent with these studies as BA patients in our study also rated themselves as more aggressive than CT subjects. This discrepancy between self-referential and objective assessments suggests that aggression is not heightened in BA but may involve abnormal self-awareness, which is further supported by the finding of the negative correlation between the self-rating scores of aggression and the objective measurement of aggression in the PSAP test in BA patients. Indeed, several psychiatric disorders, such as schizophrenia [86,87,] and addiction [88–92], are characterized by the lack of self-awareness of symptoms or even entire disorders. The lack of self-awareness of addicted patients that they are addicted to drugs or specific behaviours considerably interferes with treatment and recovery from the disorder [92,93,]. Therefore, the mechanisms that cause altered self-awareness are an important target for the treatment of addiction. Accumulating evidence suggests that deficits in the mechanisms of meta-cognition, which could be associated with neural activity of the anterior cingulate cortex and vmPFC [88,89,], may underlie this lack of self-awareness of disorders [94,95,], which was not examined in this study. A future study investigating these brain regions in the context of implicit and explicit assessments of aggression may provide further insight into the heightened aggression and abnormal self-awareness involved in BA.

In conclusion, our study has demonstrated less clear evidence of heightened aggression in some symptoms of BA, such as KM and PP. Importantly, our study is limited by a small sample size. In addition, inconsistency of subjective vs. objective rating of aggression is observed, which would be associated with compromises in self-awareness, which in turn cause a fallacy of heightened aggression, in BA. There is also a possibility that BA patients may still exhibit heighted aggression similar to patients with antisocial personality disorders, who exhibit higher aggression than healthy subjects in the PSAP task only when they are given alcohol [49]. Thus, BA could still be associated with heightened aggression, and further investigations are required. These findings suggest that behavioural therapy to manage aggression and violent behaviours, such as aggression control therapy [23], may not always be useful in BA; however, similar to drug addiction [92,93,], behavioural therapy for managing self-awareness may be helpful for the treatment of BA.
